# In the national epidemiological bulletins – a selection from recent issues

**DOI:** 10.2807/1560-7917.ES.2017.22.50.171214-1

**Published:** 2017-12-14

**Authors:** Eurosurveillance editorial team

**Affiliations:** 1 European Centre for Disease Prevention and Control (ECDC), Stockholm, Sweden

**Keywords:** infectious diseases, public health, Europe

**Healthcare-associated infections and antibiotic resistance**

Properties, frequency and distribution of vancomycin-resistant enterococci (VRE) in Germany - Update 2015/2016

Epidemiologisches Bulletin 46, 2017

16 November 2017, Germany

https://www.rki.de/DE/Content/Infekt/EpidBull/Archiv/2017/Ausgaben/46_17.pdf?__blob=publicationFile

 

**Food- and waterborne diseases**

*Campylobacter* enteritis - risk factors and sources of infection in Germany

Epidemiologisches Bulletin 44, 2017

2 November 2017, Germany

https://www.rki.de/DE/Content/Infekt/EpidBull/Archiv/2017/Ausgaben/44_17.pdf?__blob=publicationFile

 

Surveillance of *Listeria monocytogenes* in the Netherlands, 2016

Infectieziekten Bulletin 2017; 28(8)

17 October 2017, the Netherlands

http://www.rivm.nl/Documenten_en_publicaties/Algemeen_Actueel/Uitgaven/Infectieziekten_Bulletin/Jaargang_28_2017/Oktober_2017/Inhoud_oktober_2017/Surveillance_van_Listeria_monocytogenes_in_Nederland_2016

 

**Hepatitides**

Hepatitis A 2015-2016

EPI-NEWS 47, 2017

22 November 2017, Denmark

https://www.ssi.dk/Aktuelt/Nyhedsbreve/EPI-NYT/2017/Uge%2047%20-%202017.aspx

 

**Vaccine-preventable diseases**

Meningococcal disease 2016

EPI-NEWS 49, 2017

6 December 2017, Denmark

https://www.ssi.dk/Aktuelt/Nyhedsbreve/EPI-NYT/2017/Uge%2049%20-%202017.aspx

 

Measles outbreak 2017

Epi-Insight 2017;18(12)

December 2017, Ireland

http://ndsc.newsweaver.ie/epiinsight/5731j3gwddpimcmkeer4wk?a=2&p=52679956&t=17517804

 

A polio-free world?

EPI-NEWS 48, 2017

29 November 2017, Denmark

https://www.ssi.dk/Aktuelt/Nyhedsbreve/EPI-NYT/2017/Uge%2048%20-%202017.aspx

 

Laboratory confirmed cases of measles, mumps and rubella, England: July to September 2017Health Protection Report; 11(42)

24 November 2017, United Kingdom

https://www.gov.uk/government/uploads/system/uploads/attachment_data/file/662499/hpr4217_mmr.pdf

 

Special issue: Vaccination of young children: Data for a better understanding of public action

Bulletin épidémiologique hebdomadaire

19 October 2017, France

http://invs.santepubliquefrance.fr/Publications-et-outils/BEH-Bulletin-epidemiologique-hebdomadaire/Archives/2017/BEH-hors-serie-Vaccination-des-jeunes-enfants-des-donnees-pour-mieux-comprendre-l-action-publique

 

Invasive pneumococcal disease and adherence to pneumococcal vaccination in the childhood vaccination programme 2016

EPI-NEWS 41, 2017

11 October 2017, Denmark

https://www.ssi.dk/Aktuelt/Nyhedsbreve/EPI-NYT/2017/Uge%2041%20-%202017.aspx

 

**Respiratory diseases**

 

Legionnaire’s disease 2016

EPI-NEWS 45, 2017

8 November 2017, Denmark

https://www.ssi.dk/Aktuelt/Nyhedsbreve/EPI-NYT/2017/Uge%2045%20-%202017.aspx

 

Enhanced Surveillance of Mycobacterial Infections (ESMI) in Scotland: 2017 tuberculosis annual report for Scotland

HPS Weekly Report 2017; 51(43)

31 October 2017, Scotland

http://www.hps.scot.nhs.uk/documents/ewr/pdf2017/1743.pdf

 

Influenza activity in France, season 2016-17

Bulletin épidémiologique hebdomadaire, 22

10 October 2017, France

http://invs.santepubliquefrance.fr/beh/2017/22/pdf/2017_22.pdf

 

Hygiene and prevention practices of respiratory infections during the winter months: results from the 2016 Health Barometer, France

Bulletin épidémiologique hebdomadaire, 22

10 October 2017, France

http://invs.santepubliquefrance.fr/beh/2017/22/pdf/2017_22.pdf

 

Perceptions and behaviors of people aged 65 to 75 towards seasonal flu vaccination in France, 2016

Bulletin épidémiologique hebdomadaire, 22

10 October 2017, France

http://invs.santepubliquefrance.fr/beh/2017/22/pdf/2017_22.pdf

 

Method to increase vaccination coverage of healthcare workers for seasonal influenza in health care institutions

Vlaams Infectieziektebulletin; 2/2017

October 2017, Belgium

https://www.zorg-en-gezondheid.be/sites/default/files/atoms/files/VIB%202017-2_HRES_0.pdf

 

**Sexually transmitted diseases**

Special edition: World AIDS Day, December 1, 2017

Bulletin épidémiologique hebdomadaire, 29-30

28 November 2017, France

http://invs.santepubliquefrance.fr/beh/2017/29-30/pdf/2017_29-30.pdf

 

HIV infection and AIDS: Quarterly report to 30 September 2017

HPS Weekly Report 2017; 51(47)

28 November 2017, Scotland

http://www.hps.scot.nhs.uk/documents/ewr/pdf2017/1747.pdf

 

Estimation of the number of new HIV infections and the total number of people living with HIV in Germany

Epidemiologisches Bulletin 47, 2017

23 November 2017, Germany

https://www.rki.de/DE/Content/Infekt/EpidBull/Archiv/2017/Ausgaben/47_17.pdf?__blob=publicationFile

 

Cluster of cases of multi-drug resistant *Shigella* notified amongst MSM, June 2017

Epi-Insight 2017;18(11)

November 2017, Ireland

http://ndsc.newsweaver.ie/epiinsight/le4wjue46ls?a=2&p=52547623&t=17517804

 

HSE recommends antiretroviral therapy for all people living with HIV attending HIV services in Ireland

Epi-Insight 2017;18(10)

October 2017, Ireland

http://ndsc.newsweaver.ie/epiinsight/t8q985ffg1610gkzp9yxn5?a=2&p=52412028&t=17517804

 

HIV in Ireland 2016

Epi-Insight 2017;18(10)

October 2017, Ireland

http://ndsc.newsweaver.ie/epiinsight/t8q985ffg1610gkzp9yxn5?a=2&p=52412028&t=17517804

 

Genital chlamydia and gonorrhoea infection in Scotland: laboratory diagnoses 2007 – 2016

HPS Weekly Report 2017; 51(38)

26 September 2017, Scotland

http://www.hps.scot.nhs.uk/documents/ewr/pdf2017/1738.pdf

 

Gonococcal antibiotic surveillance in Scotland (GASS): prevalence, patterns and trends in 2016

HPS Weekly Report 2017; 51(38)

26 September 2017, Scotland

http://www.hps.scot.nhs.uk/documents/ewr/pdf2017/1738.pdf

 

**Zoonoses and vector-borne diseases **

Surveillance of human hantavirus infections in metropolitan France, 2012-2016

Bulletin épidémiologique hebdomadaire, 23

24 October 2017, France

http://invs.santepubliquefrance.fr/beh/2017/23/pdf/2017_23.pdf

 

Zoonotic disease in Scotland

HPS Weekly Report 2017; 51(42)

24 October 2017, Scotland

http://www.hps.scot.nhs.uk/documents/ewr/pdf2017/1742.pdf

 

Infection with TBE virus in Denmark 2013-2016

EPI-NEWS 40, 2017

4 October 2017, Denmark

https://www.ssi.dk/Aktuelt/Nyhedsbreve/EPI-NYT/2017/Uge%2040%20-%202017.aspx

 

**Other**

** **

Special edition: Mayotte: epidemiological data for the assessment and prevention of health risks

Bulletin épidémiologique hebdomadaire, 24-25

31 October 2017, France

http://invs.santepubliquefrance.fr/beh/2017/24-25/pdf/2017_24-25.pdf

 

Reportable diseases in the summer of 2017Epi-Ice 10(4), 2017

October 2017, Iceland

https://www.landlaeknir.is/servlet/file/store93/item33182/EPI-ICE_October_2017.pdf

 

Outbreaks in the summer of 2017Epi-Ice 10(4), 2017

October 2017, Iceland

https://www.landlaeknir.is/servlet/file/store93/item33182/EPI-ICE_October_2017.pdf

